# Utilization of the peroxidase-like activity of silver nanoparticles nanozyme on *O*-phenylenediamine/H_2_O_2_ system for fluorescence detection of mercury (II) ions

**DOI:** 10.1038/s41598-022-10779-8

**Published:** 2022-04-28

**Authors:** Mohamed A. Abdel-Lateef

**Affiliations:** Department of Pharmaceutical Analytical Chemistry, Faculty of Pharmacy, Al-Azhar University, Assiut Branch, Assiut, 71524 Egypt

**Keywords:** Analytical chemistry, Catalysis, Nanoparticles

## Abstract

Polyvinylpyrrolidone stabilized silver nanoparticles (PV-AgNPs) were synthesized from AgNO_3_/trisodium citrate and with the assistance of microwave energy. The synthesized PV-AgNPs were found to own an actual peroxidase mimicking activity. This catalytic activity can oxidize the non-fluorescence reagent (o-phenylenediamine) to a high fluorescence reaction product (2,3-diaminophenazine). The reaction product exhibited a fluorescence emission at 563 nm upon the excitation at 420. Among many metals, only mercury (II) ions can inhibit the catalytic activity of PV-AgNPs nanozyme. Accordingly, the fluorescence intensity of the reaction product has been successfully quenched. This quenching effect in the fluorescence intensity was directly proportional to the concentration of mercury (II). Depending on this finding, a simple, cost-effective, and selective spectrofluorimetric approach has been designed for mercury (II) detection in water samples. The linear relationship between the inhibition in fluorescence intensity and mercury (II) concentration was found in 20–2000 nM with a detection limit of 8.9 nM.

## Introduction

Mercury metal is one of the most known poisonous and widely spread heavy metals due to its harmful effects upon accumulation in the human body^1^. It is widely distributed in the soil, atmosphere, and seawater through human activities and natural phenomena, causing serious results on most living organisms and the environment^2^. The main contamination sources by mercury (II) for surface waters and wastewater are Chlor-alkali production, paper and pulp, oil refinery, batteries, and paint manufacturing processes in industries^3^. Due to its high affinity for thiol groups found in enzymes and proteins, mercury can accumulate in human body tissues and vital organs, producing toxic and damaging human health even in low quantities^4^. Some chronic and acute symptoms and signs generated by the inorganic mercury toxicity are as follows: mouth inflammation; thirst; metallic taste; nausea; excessive salivation; kidney degeneration, and tremor^5^.

The selectivity and the sensitivity of the utilized chemosensory for detecting mercury (II) ion in water samples is an essential demand. Therefore, the utilized sensor should be characterized by simplicity, low cost, high sensitivity, and adequate selectivity, which can be sensing mercury (II) ions in aqueous samples at the nanomolar level and without interferences from the presences of other metals ions.

Nanoparticles are widely applied as sensors for detecting environmental pollutants^6–9^. Many researchers are interested in silver nanoparticles due to their unusual optical features, SPR band, and ultrasmall size^10–13^. Silver nanoparticles are also widely employed in the sensor, textile industries, and food storage due to their excellent conductivity and catalytic activity^10–15^. The distinctive, unique enzyme-like activity of metals nanoparticles has attracted interest in the catalysis of numerous chemical reactions and metals analysis applications^16^.

There are many merits for using nanoparticles as an enzyme mimics/artificial enzyme over natural enzymes that focus on the absence of the inherent hindrances of the natural enzyme. These hindrances include time-consuming, tedious, availability of the natural resources and expensive purification process; sensitivity towards the elevated temperatures, rigorous storage conditions, sensitivity for the alkaline and the acidic pH conditions and proteases, leading to decreased stability lowering the shelf life^17,18^. Enzyme mimics inorganic nanoparticles are afforded with some features, including low cost, high stability, resistance to the high concentrations of the substrate, ease of storage process, and ease of synthesis^19–21^.

In general, nanoparticles of noble metals (such as gold, silver, platinum, and palladium) exhibit appealing physicochemical features that are dependent on their form and size^6,16^. For example, the peroxidase-like catalytic activity of gold nanoparticles has been utilized for the colorimetric sensing of mercury (II) and lead ions in water samples^22,23^. Furthermore, the catalytic activity of platinum nanoparticles has been utilized to detect mercury (II) ions in water samples^24,25^. Also, the catalytic activity of silver nanoparticles has been employed for visual colorimetric detection of protein and as a resonance Rayleigh scattering sensor for mercury (II) ions detection^26–28^. The catalytic activity of these nanomaterials depends on their sizes, known as the “size effect”; for instance, the great catalytic activity for gold nanoparticles can be observed with nano sizes smaller than 5.0 nm ^16,29,30^. For that reason, a lot of efforts have been devoted to reducing the size of the synthesized nanoparticles^31^.

The reported studies prove that using polyvinylpyrrolidone surfactant to prepare Ag-NPs produces tiny nano sizes below 10 nm and stabilizes the formed nanoparticles for a long period^32,33^. Furthermore, microwave irradiation energy over conventional heating causes uniform and rapid heating to the solution. It thus produces homogeneous nucleation sites in the solution and growth conditions, causing monodispersed nanoparticles in a short time^34^. Moreover, microwave irradiation can be afforded good particle size distribution and smaller particle sizes to synthesize silver nanoparticles^35^.

Fluorescence-spectrometer is a highly sensitive analytical technique that usually offers great selectivity without losing precision^36–38^. Designing a fluorescent sensor for mercury (II) ions detection relying on the peroxidase-like property of silver nanoparticles has not been investigated yet. Therefore, this work aims to use the catalytic activity of the smaller size polyvinylpyrrolidone stabilized silver nanoparticles as a nanozyme for the fluorescence detection of mercury (II) ions.

## Materials, instruments, and methods

### Materials

*O*-Phenylenediamine, polyvinylpyrrolidone, and silver nitrate have been produced by Sigma-Aldrich Chemical Co (Steinheim, Germany).

Aluminum nitrate, barium chloride, cadmium chloride, chromium chloride, cobalt nitrate, calcium chloride, magnesium chloride, hydrogen peroxide, mercuric chloride, nickel nitrate, sodium chloride, potassium chloride, and zinc nitrate have been produced by El-Nasr chemical Co. (Cairo, Egypt). Trisodium citrate has been produced by Fisher Scientific Co. (Leicestershire, UK). Ultrapure water was utilized in all experimental steps.

### Instruments

The fluorescence spectra were performed on an FS2 fluorescent spectrometer (Scinco, Korea). The morphology of the prepared silver nanoparticles has been characterized by JSM 5400 LV SEM (JEOL, Tokyo, Japan). The nano size, poly dispersion index, and the quality of the prepared silver nanoparticles have been characterized by ZEN 1690 (Malvern Instruments, Malvern, UK). SM-2000MW microwave oven (Smart Co., China) was utilized for heating process.

### Synthesis of silver nanoparticles using microwave energy

0.2% w/v PVP solution, 10 mM trisodium citrate solution and 10 mM silver nitrate solution were simultaneously poured into a 250 mL flask in the ratio of 0.5:1:1 and mixed by magnetic stirring for 3 min. The flask has been heated for about 12 min at 90 °C by microwave irradiation. The formation of polyvinylpyrrolidone silver nanoparticles (PVP-AgNPs) can be evidenced by the transformation of the colorless solution to a yellowish-green colloidal state.

### Detection of mercury (II) ion

In a series of calibrated flasks (10 mL), suitable volumes of mercury (II) solutions (in the range of 100 nM to 20 µM) and 800 µL of PVP-AgNPs solution were poured, incubated for 2 min and followed by the addition of 800 µL from *O*-phenylenediamine (prepared by dissolving 0.108 g in 100 mL water) solution. Then, 400 µL of 3% w/v hydrogen peroxide solution was added into the content, and the content was vortexed for 1 min. After incubation for 15 min at room temperature, the volume has been completed to a 10-mL by deionized water. The blank solution has been simultaneously prepared by the same steps with omitting the addition of mercury (II) solution. The quenching in the fluorescence intensity of the blank solution upon the addition mercury (II) was measured at the ${\lambda }_{emission}$ of 563 nm, upon the ${\lambda }_{excitation}$ of 420 nm. The specificity of the suggested method has been checked by the addition of different metal ions solutions at the concentration of 10 µM instead of mercury (II) ion in the abovementioned procedures.

### Detection of mercury (II) in different water samples using PVP-AgNPs

Tap water and bottled water samples were collected from our laboratory and a local establishment. The collected samples were spiked with different known concentrations of mercury (II). Then after, the water samples were filtered utilizing a 0.45 μm syringe filter to discard any particulates matter. Finally, the abovementioned general analytical assay was followed.

## Results and discussion

### Characterization and peroxidase mimicking activity of PVP-AgNPs

**Figure 1:**
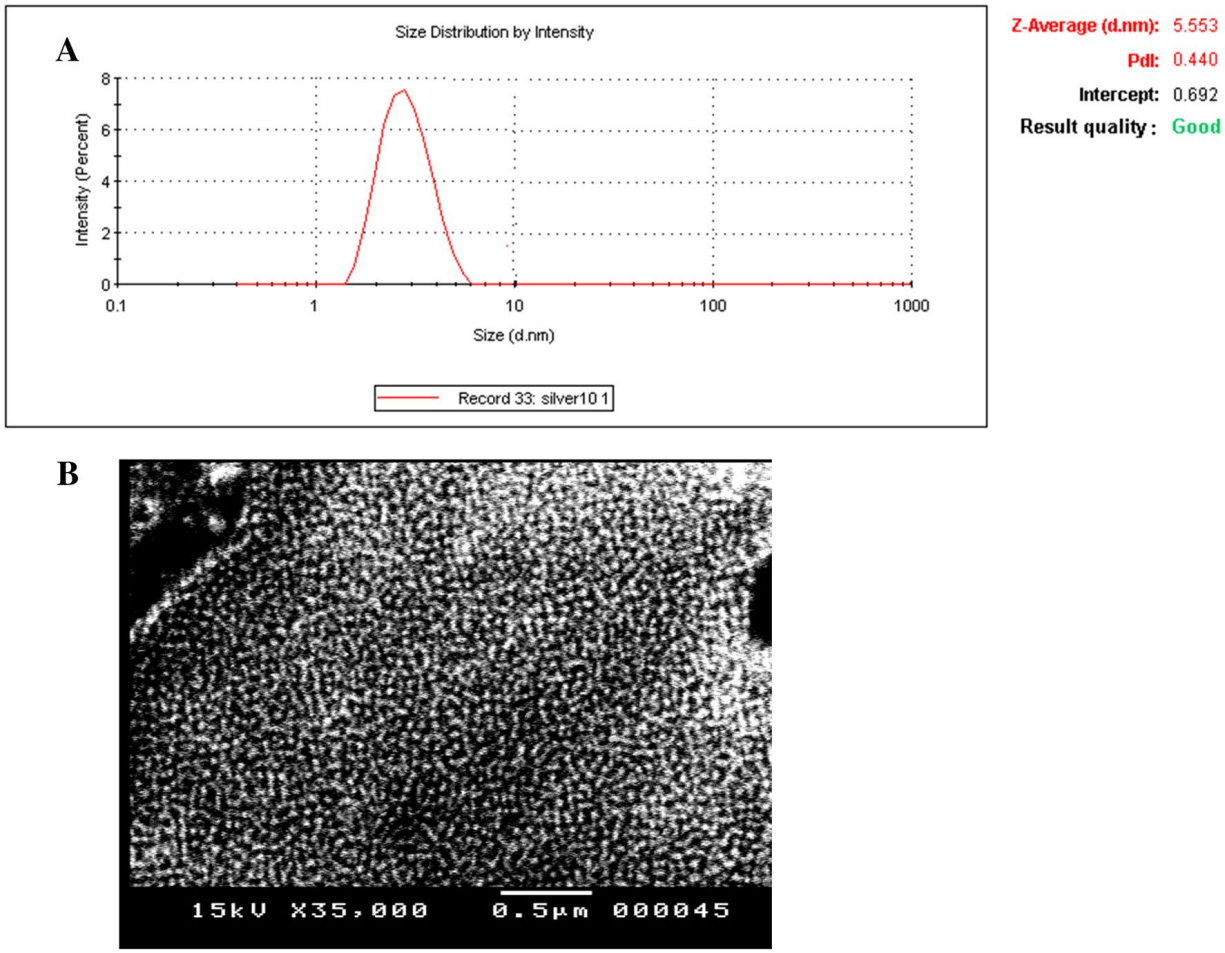
(**A**) Characterization size, PdI value and quality of the synthesized PVP Ag-NPs by zeta-sizer device; (**B**) Characterization of the morphology of the synthesized PVP Ag-NPs by SEM device.

The morphology and elemental characteristics [particle size, polydispersity index (PDI), size uniform] of PVP-AgNPs were examined using SEM device and zeta-sizer device, respectively. Figure 1 shows the SEM image of PVP-AgNPs, which refers to the spherical-rod-like shape of the synthesized nanomaterial. The measured size of the synthesized PVP-AgNPs was 5.5 nm with uniform size, good quality, and a low polydispersity index value of 0.440 Fig. 1A. The size of nanoparticles is the main factor responsible for their catalytic activity^39^. Generally, the catalytic performance of the silver nanoparticles is inversely proportional to the nanosize of their particles^40^. In the current study, the determined size of the prepared PVP-AgNPs is very small (5.5 nm), which refers to their superior catalytic activity.

**Figure 2:**
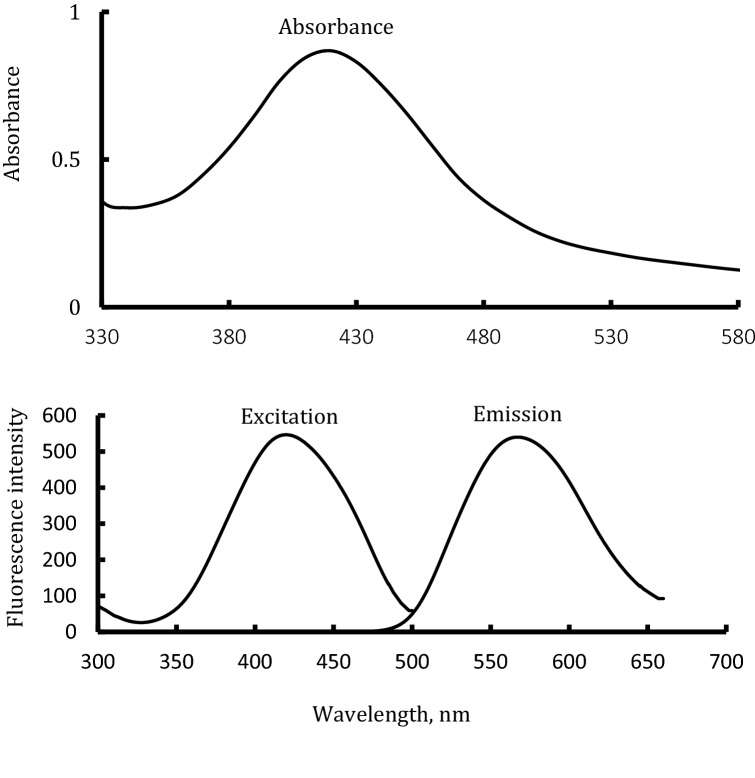
Examining the peroxidase mimicking activity for the prepared PVP-Ag-NPs by forming the characterized absorption and fluorescence (excitation/emission) spectra of OPDA reaction product (2,3-phenazinediamine).

*O*-Phenylenediamine (OPD) is one of the typical substrates utilized to investigate the peroxidase-like activity of the nanoparticles^25,41^. OPD (colorless and non-fluorescent) has been oxidized by peroxidase mimicking activity of certain nanoparticles to 2,3-phenazinediamine (colored and fluorescent)^25^. Herein, the peroxidase mimicking activity of the prepared PVP-AgNPs has been examined by the fluorescence technique and the spectrophotometric technique using OPD/H_2_O_2_ system. Practically, the catalytic activity of the prepared PVP-AgNPs has been spectrophotometrically confirmed by the appearance of characteristic absorbance peak at λ_max_ = 420 nm, Fig. 2. Furthermore, it has been fluorometrically evidenced by the existence of a distinct fluorescence peak at λ_emission_ = 563 upon λ_excitation_ = 420, Fig. 2. Moreover, it was found that only the mixture of PVP-AgNPs/OPD/H_2_O_2_ exhibited this fluorescence behavior. In contrast, neither one of the PVP-AgNPs/OPD mixture, OPD/H_2_O_2_ mixture, and PVP-AgNPs/H_2_O_2_ mixture has produced any fluorescence character at the same conditions. These absorbance peak and fluorescence emission peak values are matching to those of 2,3-phenazinediamine that were reported in studies of literature^25^.

### Fabrication and design of mercury (II) fluorescence detection sensing system

It is well known that the colored 2,3-phenazinediamine, which possesses a distinctive fluorescent behavior at λ_emission_ = 563 nm/λ_excitation_ = 420 nm, is the oxidized product of o-phenylenediamine. Initially, the colorless solution changed from non-fluorescent to a bright fluorescence yellow solution when the PVP-AgNPs were added to the o-phenylenediamine/H_2_O_2_ system. In this reaction, the prepared PVP-AgNPs owned peroxidase mimicking activity that can catalyze the oxidation of o-phenylenediamine with H_2_O_2_ to yield 2,3-phenazinediamine as the main reaction product.

**Figure 3:**
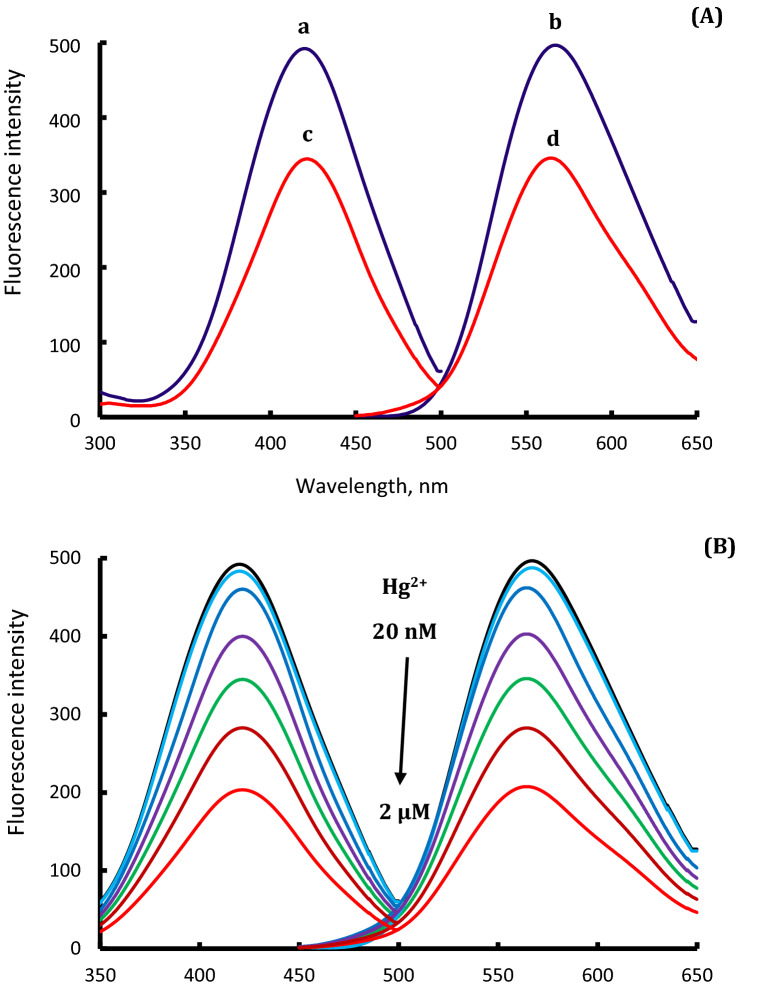
(**A**) for fluorescence (excitation and emission) spectra of OPD/H_2_O_2_/PVP-Ag-NPs system (a, b, respectively) and after their quenching by 1.0 µM of Hg^2+^ (c, d), while (**B**) for fluorescence spectra of OPD/H_2_O_2_/PVP-Ag-NPs system in the presence of various concentrations of Hg^2+^.

**Scheme 1:**
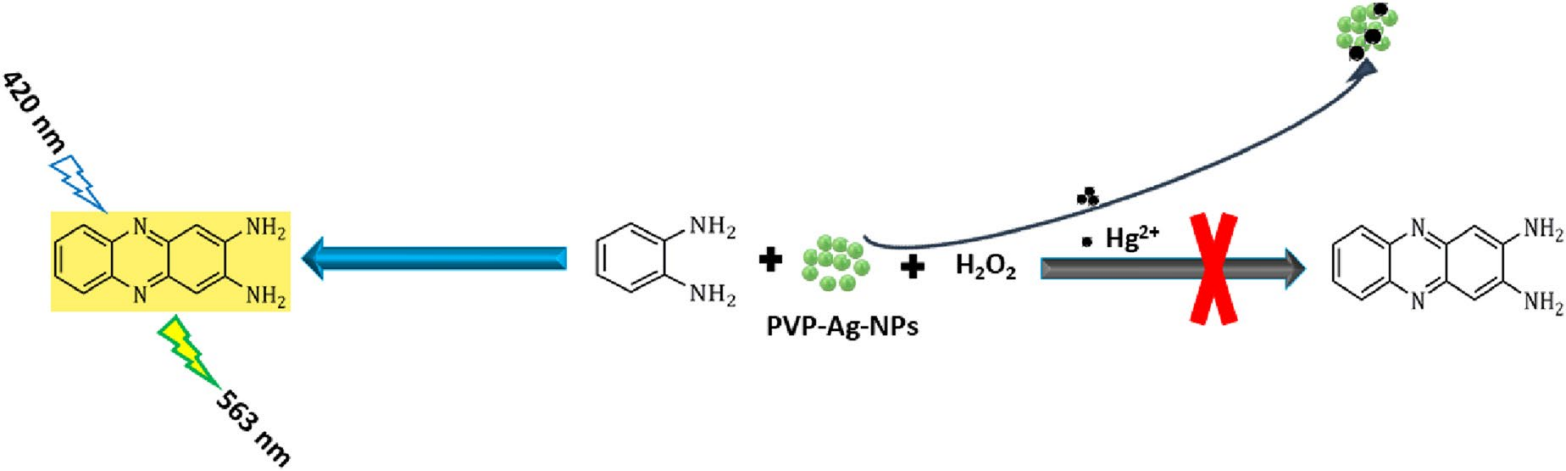
Schematic elucidation for the detection of mercury (II) by spectrofluorimetric technique.

Mercury has a unique advantage over the rest of the elements through its ability to form an amalgam with certain elements such as gold, platinum, and silver^24,25,27,28,42,43^. Therefore, in this study, the formation of Ag–Hg amalgam produces an effective inhibition for the catalytic activity of PVP-AgNPs, combined with changing their surface properties. This catalytic inhibition effect of PVP-AgNPs prevents the transformation of the OPD/H_2_O_2_ system to 2,3-phenazinediamine. Accordingly, a quenching action on the fluorescence intensity of the solution upon the addition of mercury (II) in comparison with the fluorescence intensity of blank sample (without Hg^2+^), Fig. 3. Therefore, the selective determination of mercury (II) in a certain linear concentration range was achieved. The sensing mechanism of mercury (II) is illustrated in Scheme 1.

### Optimization of the sensing system and fluorescent readout assay of mercury (II)

**Figure 4:**
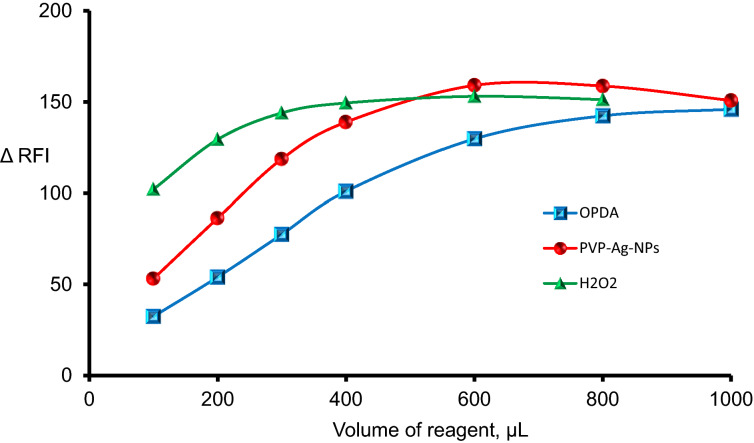
Investigating the volume of reagents for Hg^2+^ (1.0 µM) detection by the suggested method.

The reaction conditions involved the volume of H_2_O_2_, reaction time, the volume of OPDA, and the volume of PVP-Ag-NPs were examined and optimized to find the optimum conditions for analysis. The sensing system was incubated with mercury (II) for 15 min and the optimum conditions for the quenching in the fluorescence intensity of the solution (in compared to the blank solution) was obtained with 800 µL of PVP-AgNPs suspension, 800 µL of OPD solution, and 400 µL of 3% w/v hydrogen peroxide solution, Fig. 4. This quenching effect in the fluorescence intensity of the solution is directly proportional with the concentration of mercury (II).

**Figure 5:**
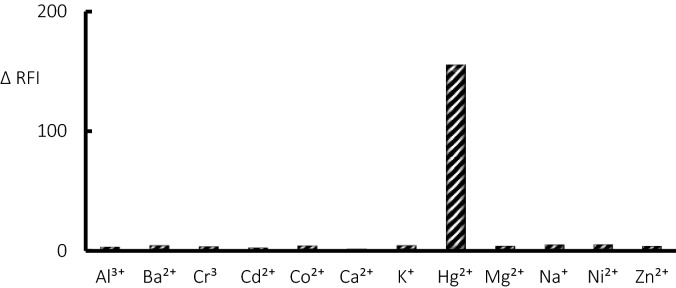
Examining the selectivity of the utilized system (OPD + H_2_O_2_ + PVP-Ag-NPs) for the detection of Hg^2+^ ion (1 µM) in the presence of common metals ions (10 µM).

The linear relationship between the quenching of fluorescence emission at 563 nm and mercury (II) concentration was established in the ranging of 20 nM to 2 μM with the regression equation of $y=0.1352x +13.51$, R^2^ value of 0.998, and LOD value of 8.9 nM (S/N = 3, where N represents noise and S represents sensitivity). The statistical parameters for detecting Hg^2+^ by the fluorescence methodology are presented in Table 1. Other common metals such as Al^3+^, Ba^2+^, Ca^2+^, Cd^2+^, Co^2+^, Cr^3+^, K^+^, Mg^2+^, Ni^2+^, Na^+^, and Zn^2+^ have been tested by the current fluorescent methodology to investigate the selectivity of the design sensing system. It was found that there is no obvious effect in the emission intensity of PVP-AgNPs/OPD/H_2_O_2_ system have been detected upon adding of any metals from mentioned metals at higher concentration level (tenfold excess in compared to mercury (II)). In contrast, the emission intensity of PVP-AgNPs/OPD/H_2_O_2_ has been significantly decreased in the presence of mercury (II) which refers to the good selectivity for the fabricated system (Fig. 5). The explanation for the perfect selectivity of the proposed method for mercury (II) may be attributed to the formation of Ag–Hg amalgam through a specific interaction between silver nanoparticles and mercury (II) ion^42^.

**Table 1:** Analytical parameters for the proposed fluorescence method.

Parameter	Spectrofluorimetric assay
Linear range (nM)	20–2000
Standard error	5.41
Intercept	13.35
Standard error of intercept	3.42
Slope	0.1353
Standard error of slope	0.003
Correlation coefficients (r)	0.9989
Determination coefficients (r^2^)	0.9979
Number of determinations	6

### Fluorescence detection of mercury (II) ions in tap and bottled water samples

The applications of PVP-AgNPs for spiked samples experiments were performed with tap water samples and bottled water samples. To evaluate the practicality for applying the fluorescent methodology in tap water samples and bottled water samples have been spiked with mercury (II) at different concentration levels and tested by the suggested methodology. The data in Table 2 refers to the good recovery and SD values for determining mercury (II) ions by the current method. These SD and recovery values evidenced the validity of accuracy and precision of the presented methodology for mercury (II) detection in tap water samples and bottled water samples.

**Table 2:** Results for the detection of mercury (II) in tap and bottled water samples.

Sample	Added Hg^2+^ (nM)	Found Hg^2+^ (nM)	Recovery ± SD
Tap water	500	510.54	102.11 ± 1.54
Tap water	1000	979.38	97.94 ± 2.22
Tap water	2000	2057.48	102.87 ± 2.21
Bottled water	500	513.75	102.75 ± 2.08
Bottled water	1000	1013.38	101.34 ± 1.97
Bottled water	2000	1980.61	99.03 ± 2.04

## Conclusions

In this study, the microwave irradiation energy was utilized to assist the synthesis of polyvinylpyrrolidone stabilized silver nanoparticles with very small nano sizes. The prepared silver nanoparticles showed distinctive peroxidase activity behavior. Based on the inhibition effect of mercuric (II) ions towards this peroxidase activity of the prepared silver nanoparticles, a fluorescent methodology with a highly sensitive and extremely selective response towards mercuric (II) ions detection was established. Under the optimum conditions, the provided assay exhibited a detection limit (LOD) of 8.9 nM with a linear range of 20–2000 nM. The current methodology proffered some advantages regarding to the greenness of synthesis, tiny particle size, good stability, and uniformity, using smaller concentration from the nanozyme, and easiness of the detection.
